# A COMMUNITY AND TECH-BASED APPROACH FOR HYPERTENSION SELF-MANAGEMENT (COACHMAN)

**DOI:** 10.1093/geroni/igz038.269

**Published:** 2019-11-08

**Authors:** Carolyn H Still, Shirley M Moore, Abdus Sattar, Jackson T Wright Jr

**Affiliations:** 1 Case Western Reserve University, Cleveland, Ohio, United States; 2 Case Western Reserve University, Frances Payne Bolton School of Nursing, Cleveland, Ohio, United States; 3 Case Western Reserve University, School of Medicine, Cleveland, Ohio, United States

## Abstract

African American (AAs) are disproportionately affected by hypertension. Developing effective outreach programs with community partners is a major public health priority and ideal to educate, empower, and offer support to self-manage hypertension in AAs. The purpose of this pilot is to investigate the effectiveness of a community outreach program using a technology-based intervention for hypertension self-management (COACHMAN) to improve blood pressure (BP) control. Forty AAs with hypertension will be randomly assigned to COACHMAN or enhanced usual care (EUC). COACHMAN is comprised of four components: self-monitoring of BP; web-based education; nurse counseling; and training on a medication management application. The primary outcome is change in BP from baseline to 3-months. We hypothesize that participants in COACHMAN (compared to EUC) will have better BP control. Findings from this study, if confirmed, will provide knowledge to the scarce literature available on technology-based interventions appropriate to help AAs self-manage hypertension and improve BP control.

